# The complete mitochondrial genome of the bumblebee, *Bombus hypocrita sapporensis* (Insecta: Hymenoptera: Apidae) from Hokkaido Island, Japan

**DOI:** 10.1080/23802359.2018.1450673

**Published:** 2018-03-14

**Authors:** Mana Nishimoto, Misuzu Umezawa, Hisashi Okuyama, Norikuni Kumano, Tetsuro Nomura, Jun-ichi Takahashi

**Affiliations:** a Faculty of Life Sciences, Kyoto Sangyo University, Kyoto, Japan;; b Department of Agro-environmental Science, Obihiro University of Agriculture and Veterinary Medicine, Obihiro, Japan

**Keywords:** Bumblebee, Illumina sequencing, subspecies, *Bombus hypocrita*, Hokkaido

## Abstract

The complete is mitochondrial genome of the Japanese bumblebee *Bombus hypocrita sapporensis* from Hokkaido Island, Japan is analysed using next generation sequencing. The mitochondrial genome of *B. h. sapporensis* was observed to be a circular molecule of 15,835 bp. The average AT content in the *B. h. sapporensis* mitochondrial genome was 85.53%. It was predicted to contain 13 protein-coding genes (PCGs), 22 tRNA genes, and two rRNA genes, along with one A + T-rich control region. The PCGs had ATA, ATG, or ATT as the initiation codon and were terminated by the typical stop codon TAA, except for *Cytb*. All the tRNA genes typically formed a cloverleaf secondary structure, except for *trnE*, *trnF*, and *trnS1*. The molecular phylogenetic analysis indicated that the *B. h. sapporensis* from Hokkaido Island population was most similar to that of the geographically isolated *B. h. sapporensis* from Rebun Island.

The orange-tailed bumblebee in Far East Asian continent including North China was *Bombus patagiatus*, widely distributed to Russia, on the other hand, the orange-tailed bumblebee in Japan was true *Bombus hypocrita* (Williams, An, et al. 2012; Williams, Brown, et al. 2012). Williams, An, et al. (2012) and Williams, Brown, et al. (2012) indicated that orange-tailed bumblebee was a separate species in Japan and North China in mitochondrial DNA *COI* gene analyses using the DNA barcoding method. In morphological taxonomic analysis, Japanese *B. hypocrita* is classified into two subspecies, *B. hypocrita sapporensis* in Hokkaido Island, and *B. h. hypocrita* in Honshu, Shikoku and Kyushu Islands (Sakagami and Ishikawa 1969; Ito 1998; Ito and Munakata 1979), but the phylogenetic relationship of two *B. hypocrita* subspecies on Japan remains uncertain (Takahashi, Nishimoto, et al. 2016; Nishimoto et al. 2017; Takeuchi et al. 2017). Here we analysed the complete mitochondrial genome of orange-tailed bumblebee *B. h. sapporensis* from Hokkaido Island in Japan.

Adult workers of the *B. h. sapporensis* on wild flowers in the Otofuke Town (42°56′43″N 143°12′06″E), Hokkaido Islands, Japan, were collected in July 2017. The collected workers were transferred immediately to 99% ethanol for mitochondrial DNA analysis (the specimen was stored in the National Museum of Nature and Science, Japan, accession number: NSMT-I-HYM 74241). Genomic DNA isolated from males was sequenced using Illumina’s MiSeq platform (Illumina, San Diego, CA). The resultant reads were assembled and annotated using the MITOS web server (Bernt et al. 2013) and Geneious R9 (Biomatters, Auckland, New Zealand). Phylogenetic analysis was performed using the TREEFINDER (Jobb 2015) based on nucleotide sequences of the 13 protein-coding genes (PCGs).

The average AT content of the *B. h. sapporensis* mitochondrial genome was 85.53%. Similar to the *B. hypocrita* mitochondrial genomes from Honshu and Rebun Islands (Takahashi, Nishimoto, et al. 2016; Nishimoto et al. 2017), the heavy strand (H-strand) was predicted to contain nine PCGs and 13 tRNA genes, and the light strand (L-strand) was predicted to contain PCGs, nine tRNA genes, and two rDNA genes (Figure 1). The genes, *ATP8* and *ATP6*, shared 19 nucleotides, *ND4* and *ND4L* shared one nucleotide, and *ND6* and *Cytb* shared 13 nucleotide. Six PCGs of the *B. h. sapporensis* mitochondrial genome started with ATA, *ATP6*, *COIII*, *ND4*, and *Cytb* started with ATG, and *COII*, *ND5* and *ND4L* started with ATT; these starting codons have been found to be common in the other *Bombus* mitochondrial genomes (Cha et al. 2007; Hong et al. 2008; Du et al. 2015; Takahashi, Sasaki, et al. 2017; Zhao, Huang, et al. 2017; Zhao, Wu, et al. 2017). The stop codon in each of these PCGs was TAA, except for *Cytb*, which had TAG, as in other bumblebees.

**Figure 1.**
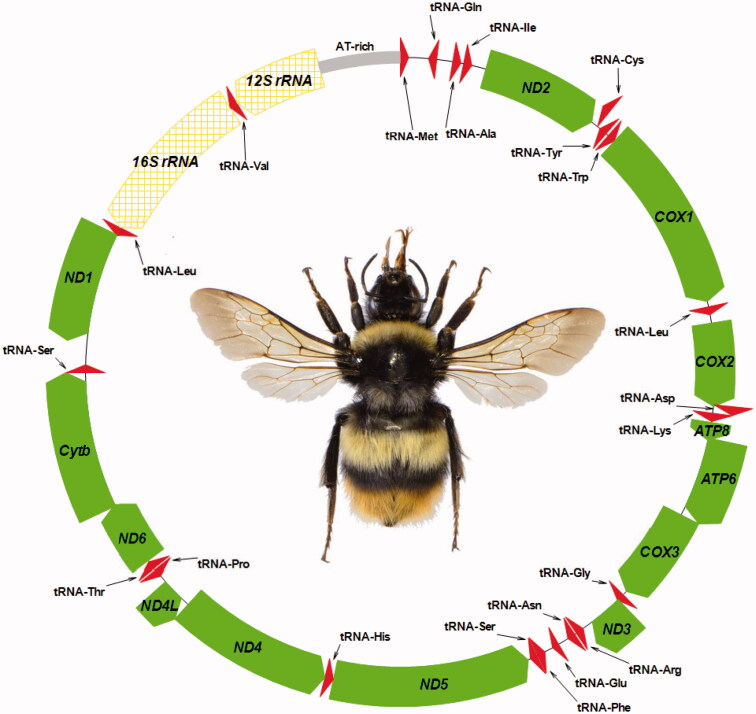
Physical map of the mitochondrial genome of *Bombus hypocrita sapporensis*. Genes illustrated on the outside of the main circle are encoded on the heavy (H) strand; genes on the inside of the circle are encoded on the light (L) strand. The 13 protein-coding genes are labelled in filled arrow (green), 22 tRNA genes are labelled in triangle (red), and 16S rRNA and 12S rRNA genes are labelled in grid pattern arrow (yellow).

Molecular phylogenetic analysis was constructed by applying 13 mitochondrial PCGs with 12 closely related taxa (Figure 2). The genetic distance and mutation site of the *B. hypocrita* in Japan taxa mitochondrial genomes were 0.0002–0.0018 and 2–20, on the other hand that of the orange-tailed bumblebee in Asia continent and the Japanese *B. hypocrita* were 0.0103–0.0107 and 113–117. The *B. hypocrita* from three Japanese Islands were shown to be a valid subspecies from the *B. hypocrita* in Asia continent by complete mitochondrial DNA sequence.

**Figure 2.**
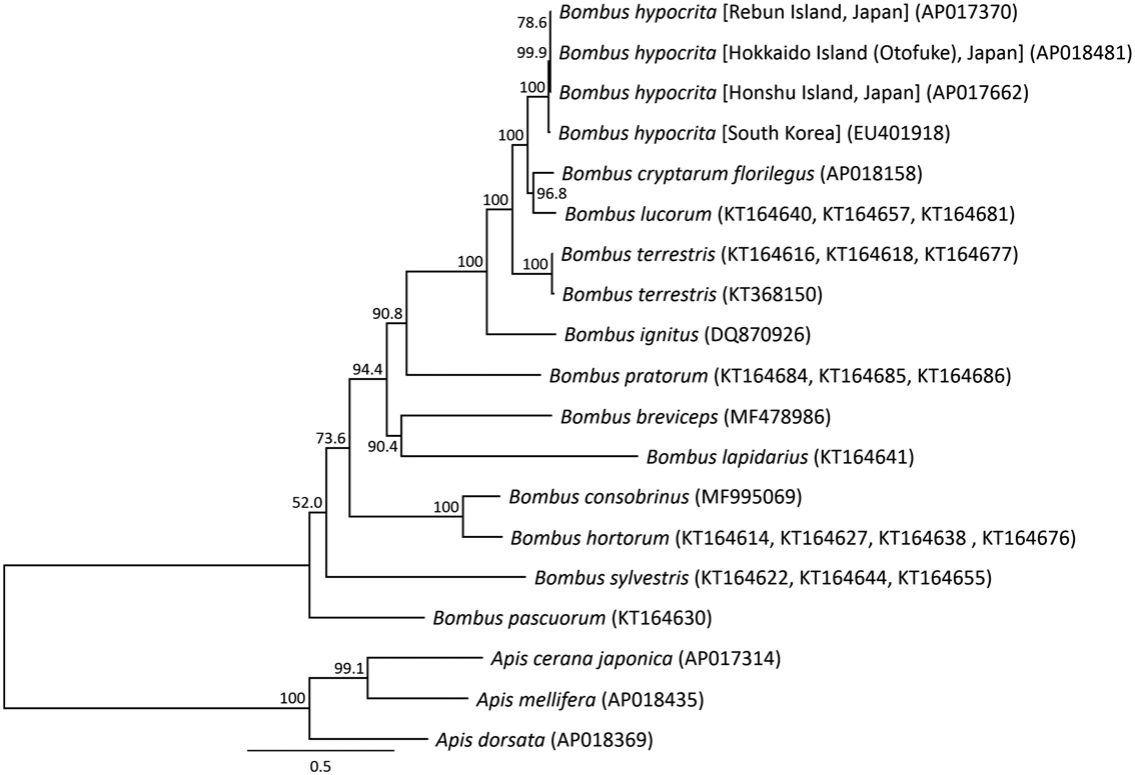
Phylogenetic relationships (maximum likelihood) among the species of the genus *Bombus* (Hymenoptera) based on the mitochondrial genome nucleotide sequence of the 13 protein-coding genes. Numbers beside the nodes are percentages of 1000 bootstrap values. The *Apis cerana* (Takahashi et al. 2016)*, Apis mellifera* (Nakagawa et al. 2018), and *Apis dorsata* (Takahashi et al. 2017) were used as outgroup. Alphanumeric in parentheses indicates the DNA database of Japan accession numbers.

## References

[CIT0001] Bernt M, Donath A, Jühling F, Externbrink F, Florentz C, Fritzsch G, Pütz J, Middendorf M, Stadler PF. 2013 MITOS: improved de novo Metazoan Mitochondrial Genome Annotation. Mol Phylogenet Evol. 69:313–319. 22982435 10.1016/j.ympev.2012.08.023

[CIT0002] Cha SY, Yoon HJ, Lee EM, Yoon MH, Hwang JS, Jin BR, Han YS, Kim I. 2007 The complete nucleotide sequence and gene organization of the mitochondrial genome of the bumblebee, *Bombus ignitus* (Hymenoptera: Apidae). Gene. 392:206–220. 17321076 10.1016/j.gene.2006.12.031

[CIT0003] Du Q, Bi G, Zhao E, Yang J, Zhang Z, Liu G. 2015 Complete mitochondrial genome of *Bombus terrestris* (Hymenoptera: Apidae). Mitochondrial DNA. 5:1–2. 10.3109/19401736.2015.1089568 26437124

[CIT0004] Hong MY, Cha SY, Kim DY, Yoon HJ, Kim SR, Hwang JS, Kim KG, Han YS, Kim I. 2008 Presence of several tRNA-like sequences in the mitochondrial genome the bumblebee, *Bombus hypocrita sapporoensis* (Hymenoptera: Apidae). Genes Genomics. 30:307–318.

[CIT0005] Ito M, Munakata M. 1979 The bumblebees in Southern Hokkaido and Northwest Honshu, with notes on Blakiston Zoogeographical Line. J Low Temp Sci B. 37:81–105.

[CIT0006] Ito M. 1998 Species and distribution of Japanese Bumblebee. Insects Nat. 33:4–7 (in Japanese).

[CIT0007] Jobb G. 2015 TREEFINDER version of March 2011, Munich. http://www.treefnder.de.

[CIT0008] Nakagawa M, Maeda M, Chikano M, Okuyama H, Murray R, Takahashi J. 2018 The complete mitochondrial genome of the yellow coloured honeybee *Apis mellifera* (Insecta: Hymenoptera: Apidae) of New Zealand. Mitochondrial DNA B. 3:66–67. 10.1080/23802359.2017.1422401 PMC7800568 33474068

[CIT0009] Nishimoto M, Okuyama H, Kiyoshi T, Nomura T, Takahashi J. 2017 Complete mitochondrial genome of the Japanese bumblebee, *Bombus hypocrita hypocrita* (Insecta: Hymenoptera: Apidae). Mitochondrial DNA B. 2:19–20. 10.1080/23802359.2016.1275849 PMC7800507 33473702

[CIT0010] Sakagami SF, Ishikawa R. 1969 Note pre ´ liminaire sur la re ´partition ge ´ographique des bourdons japonais, avec descriptions et remarques sur quelques formes nouvelles ou peu connues. J Faculty Sci Hokkaido Univ (Zool). 71:152–196.

[CIT0011] Takahashi J, Deowanish S, Okuyama H. 2017 Analysis of the complete mitochondrial genome of the giant honeybee, *Apis dorsata*, (Hymenoptera: Apidae) in Thailand. Conserv Genet Resour. doi 10.1007/s12686-017-0942-7 PMC7800543 33474166

[CIT0012] Takahashi J, Nishimoto M, Wakamiya T, Takahashi M, Kiyoshi K, Tsuchida K, Nomura T. 2016 Complete mitochondrial genome of the Japanese bumblebee, *Bombus hypocrita sapporensis* (Insecta: Hymenoptera: Apidae). Mitochondrial DNA B. 1:224–225. 10.1080/23802359.2016.1155423 PMC7800672 33473459

[CIT0013] Takahashi J, Sasaki T, Nishimoto M, Okuyama H, Nomura T. 2017 Characterization of the complete sequence analysis of mitochondrial DNA of Japanese rare bumblebee species *Bombus cryptarum florilegus*. Conserv Genet Resour. doi:10.1007/s12686-017-0832-z

[CIT0014] Takahashi J, Wakamiya T, Kiyoshi T, Uchiyama H, Yajima S, Kimura K, Nomura T. 2016 The complete mitochondrial genome of the Japanese honeybee, *Apis cerana japonica* (Insecta: Hymenoptera: Apidae). Mitochondrial DNA B. 1:156–157. 10.1080/23802359.2016.1144108 PMC7800529 33473444

[CIT0015] Takeuchi T, Takahashi M, Nishimoto M, Kiyoshi T, Tsuchida K, Nomura T, Takahashi J. 2018 Genetic structure of the bumble bee *Bombus hypocrita sapporiensis*, a potential domestic pollinator for crops in Japan. J Apic Res. 57:203–212.

[CIT0017] Williams PH, An J, Brown MJF, Carolan JC, Goulson D, Huang J, Ito M. 2012 Cryptic bumblebee species: consequences for conservation and the trade in greenhouse pollinators. PLoS One. 7:e32992. 22427924 10.1371/journal.pone.0032992 PMC3302899

[CIT0018] Williams PH, Brown MJF, Carolan JC, An J, Goulson D, Aytekin AM, Best LR, Byvaltsev AM, Cederberg B, Dawson R, et al 2012 Unveiling cryptic species of the bumblebee subgenus *Bombus s. str.* worldwide with COI barcodes (Hymenoptera: Apidae). Syst Biodivers. 10:21–56.

[CIT0019] Zhao X, Huang J, Sun C, Jiandong A. 2017 Complete mitochondrial genome of *Bombus consobrinus* (Hymenoptera: Apidae). Mitochondrial DNA B. 2:770–772. 10.1080/23802359.2017.1390422 PMC7800586 33473976

[CIT0020] Zhao X, Wu Z, Huang J, Liang C, An J, Sun C. 2017 Complete mitochondrial genome of *Bombus breviceps* (Hymenoptera: Apidae). Mitochondrial DNA B. 2:604–606. 10.1080/23802359.2017.1372710 PMC7800184 33473917

